# Integrated Magnetohydrodynamic Pump with Magnetic Composite Substrate and Laser-Induced Graphene Electrodes

**DOI:** 10.3390/polym13071113

**Published:** 2021-04-01

**Authors:** Mohammed Asadullah Khan, Jürgen Kosel

**Affiliations:** 1 Division of Physical Science and Engineering, King Abdullah University of Science and Technology (KAUST), Thuwal 23955-6900, Saudi Arabia; mohammedasadullah.khan@kaust.edu.sa; 2 Department of Automatic Control and Systems Engineering, The University of Sheffield, Sheffield S1 3JD, UK; 3 Silicon Austria Labs GmbH, Sensor Systems, Europastraße 12, 9524 Villach, Austria

**Keywords:** polyimide (PI), polydimethylsiloxane (PDMS), magnetohydrodynamic pump, lab on chip, microfluidics, magnetic composite, laser-induced graphene (LIG)

## Abstract

An integrated polymer-based magnetohydrodynamic (MHD) pump that can actuate saline fluids in closed-channel devices is presented. MHD pumps are attractive for lab-on-chip applications, due to their ability to provide high propulsive force without any moving parts. Unlike other MHD devices, a high level of integration is demonstrated by incorporating both laser-induced graphene (LIG) electrodes as well as a NdFeB magnetic-flux source in the NdFeB-polydimethylsiloxane permanent magnetic composite substrate. The effects of transferring the LIG film from polyimide to the magnetic composite substrate were studied. Operation of the integrated magneto hydrodynamic pump without disruptive bubbles was achieved. In the studied case, the pump produces a flow rate of 28.1 µL/min. while consuming ~1 mW power.

## 1. Introduction

In lab-on-chip systems, miniaturized pumps facilitate different processes such as transportation, filtration, mixing, segregation, etc. Thus, there is widespread interest in the development and deployment of such pumps for various applications. In [1], a microfluidic pump was used to study circulation in microvascular networks. A microblower-based fluidic pump was used to generate droplets with controllable sizes and chemical composition [2]. A piezoelectric titanium fluidic pump was reported in [3] for implantable medical applications. In [4], a passive ionogel pump was used to control fluid flow in a micropaper analytic device for low-cost analytical applications. A traveling wave electroosmotic pump implemented using CMOS technology is presented in [5], which is capable of pumping diluted human serum for in vitro diagnostic systems.

There are two different approaches toward producing fluid propulsion in lab-on-chip systems. The first one utilizes mechanical force generated using structures such as membranes, bellows, rotary gears, etc., to move the fluid in the system. The mechanical structures can be driven using different transduction methods such as magnetic, piezoelectric, acoustic, hydraulic, electrostatic, pneumatic, thermal, or electrostatic actuation [6,7]. The fabrication of these pumps is often complicated involving multiple masking, etching, and bonding steps. Except for rotary and peristaltic pumps, mechanical pumps produce oscillatory bidirectional flow. Thus, the output of mechanical pumps needs to be rectified using valves to obtain the monodirectional flow desirable for biofluidic applications [8]. The need to incorporate a valve alongside the pumping mechanism further increases the fabrication complexity. Mechanical pumps, due to their very nature, are prone to failure due to mechanical wear and tear. Further, the moving parts in these pumps can damage delicate biological specimens. The second approach toward fluid propulsion does away with moving parts, utilizing instead physical phenomena such as Lorentz force, electrowetting, osmotic pressure, electrostatic forces, and phase transfer to facilitate fluid motion. In general, nonmechanical pumps are easier to fabricate than their mechanical counterparts. They also scale much better to smaller device dimensions and are more reliable [8].

A pump that utilizes the Lorentz force is called magnetohydrodynamic (MHD) pump. Large-scale MHD pumps have been extensively studied as a means for propelling marine vessels [9,10,11]. Smaller-scale MHD pumps are popular for biofluidic applications, since they can be operated at much lower voltage (<10 V) compared to electrostatic and electrowetting pumps (voltages of 100 V and higher are common), which prevents electrostatic discharge problems [8].

**Figure 1:**
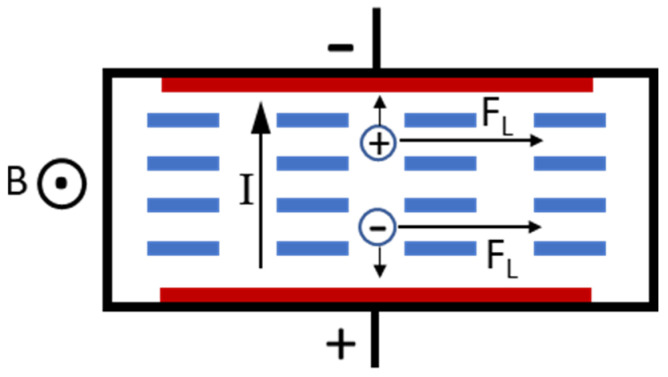
Operating principle of a typical ionic fluid magnetohydrodynamic (MHD) pump.

Figure 1 depicts the concept of a typical ionic fluid MHD pump. It consists of two electrodes to pass current through the fluid and a magnetic-flux source, which generates a magnetic field orthogonal to the electric current. When electric potential is applied to the pump, the anions get attracted to the anode and the cations to the cathode. Thus, when only electric field is applied, the ions move in the opposite directions, and thereby, there is no propulsive force on the liquid, and it is stationary. However, when a magnetic field with flux density, B is applied, an ion in the fluid with charge q and moving with a velocity v_I_ experiences the Lorentz force in a direction orthogonal to both the direction of the current as well the applied magnetic field. This force is given by

F_L_ = q · (v_I_ × B). (1)

The cations and anions are both propelled in the same direction by the Lorentz force. These ions collide with the adjacent molecules and ions, thereby imparting momentum to the fluid in a direction that is perpendicular to both the electric as well as the magnetic fields. This is the MHD effect. For an MHD pump operating with current I, flux density B, channel width w, channel depth d, and channel resistance R, it can be shown [12,13] that the flow velocity is

(2) $$v\;=\;\frac{\mathrm{IB}}{{\mathrm{Rwt}}^{2}}.$$

In order to provide the magnetic flux necessary for MHD pump operation, either an electromagnetic coil or a permanent magnet are conventionally used. Extremely high magnetic-flux density (~a few T) can be achieved using superconducting coils [10]. However, these coils need to be cooled to extremely low temperature and need sophisticated electronics to drive them [14]. As such, they are used solely in large-scale MHD pumps, such as those found in ship propulsion systems. In smaller-scale biofluidic MHD pumps, small electromagnetic coils are used, which are incapable of providing high-flux density and also suffer from high power consumption [15,16]. However, they offer certain unique benefits such as the ability to operate without bubbles by driving an MHD pump with an AC current, synchronously with an AC magnetic field [15]. Permanent magnets are popular magnetic-flux sources for small-scale MHD pumps, since they achieve a reasonably high-flux density without the need for an external power source. The rigidity of these magnets makes them ill-suited for integration with polymer-based biofluidic systems. The suitability of a NdFeB-PDMS magnetic composite for MHD pump applications was demonstrated before [17] using a prototype system. In this work, a closed-channel fluidic MHD pump is presented, which uses a magnetic composite substrate and completely eliminates the need for postprocess integration of a magnetic-flux source in MHD pumps. 

Due to the linear dependence of flow velocity on current, MHD pumps are well-suited to pump conductive fluids. Due to their extremely low resistance, liquid metals like mercury and gallium have been used as the pumping media for MHD pumps. However, these are not relevant for biofluidic applications, which deal with fluids such as blood, saliva, urine, and sea water that are saline in nature. Passing current through saline fluids causes electrolysis, resulting in bubble formation, which causes two issues (i) chlorine gas at the anode (due to oxidation of chloride ions) that is highly corrosive and (ii) gas bubbles that are highly disruptive and antagonistic to smooth flow in closed-channel devices. Noble metal electrodes such as gold [18] or platinum [19] can be utilized to mitigate the corrosion issue. However, they are expensive, and problems occur with adhesion, especially in case of flexible devices. Further, using noble metals does not address the problem of bubble-induced flow disruption. In [19], a frit structure has been utilized to isolate bubbles from the main pumping channel. Another method to mitigate these issues is to use a redox solution as the pumping medium, as demonstrated in [18]. However, this redox solution has to be properly coupled to the fluid that needs to be pumped to ensure satisfactory performance. Laser-induced graphene (LIG), manufactured by laser treatment of polyimide [20], is resistant to corrosion by chlorine and is a promising electrode material for MHD pumps [17]. However, integration of this material in closed-channel systems has not been demonstrated previously. Herein, we transfer the LIG from the polyimide to the magnetic composite facilitating integration in closed-channel lab-on-chip systems. At low current, the rate of gas evolution from the electrode is lower than the rate of dissolution of the gas in the pumping medium [21,22]. Thus, a low-drive current scheme is used to avoid disruptive bubble formation in the closed-channel integrated MHD pump.

## 2. Materials and Methods

The first step in the fabrication of the MHD pump is the formation of the LIG electrodes. This is done by treating a 127 µm thick commercial polyimide film DuPont™ Kapton^®^ HN) with CO_2_ laser at a power of 3.5 W and a speed of 92 mm/s. Ambient air was continuously circulated near the laser using the integrated nozzle of the laser printing system, which is essential for formation of good quality LIG electrodes [23]. 

PDMS (Sylgard^®^ 184) is prepared by mixing the prepolymer and curing agent in 10:1 ratio by weight. The magnetic composite is formed by mixing NdFeB micropowder (Molycorp MQP-16-7FP, mean particle size 5 µm) in a 3:1 weight ratio in uncured PDMS (75% by weight). The uncured magnetic composite is then cast onto the LIG electrodes. This is then subject to vacuum desiccation, which allows the magnetic composite to permeate through the porous LIG. The composite is then thermally cured at 90 °C for 60 min. Subsequently, the PI is carefully peeled off the magnetic composite, transferring the LIG electrodes onto the magnetic composite [24]. The magnetic and mechanical characteristics of the composite material can be found in the Supplementary Materials.

A PMMA mold is fabricated by laser patterning for facilitating the fabrication of the PDMS channel. Pure PDMS is poured into the mold and cured thermally at 90 °C for 60 min forming the channel, which has a depth of 2 mm and a width of 5 mm. Prior to release, the mold with the cured PDMS in it is placed in a refrigerator at 4 °C for 10 min. Since, PDMS has a higher coefficient of expansion than PMMA [25,26], it shrinks more than the mold, which facilitates easier release of the PDMS. 

**Figure 2:**
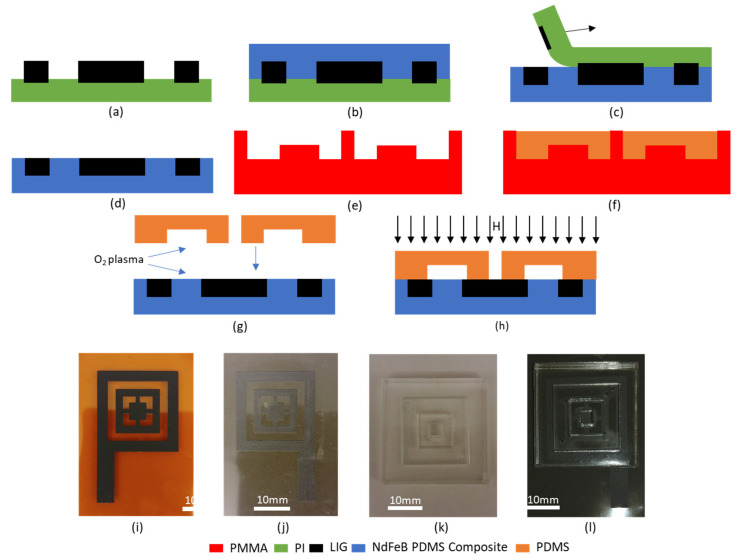
Fabrication of integrated MHD pump (**a**) laser-induced graphene LIG electrodes on PI, (**b**) NdFeB-PDMS composite casting, (**c**) LIG transfer, (**d**) LIG on magnetic composite, (**e**) PMMA mold fabrication (**f**), PDMS casting, (**g**) oxygen plasma treatment and bonding, (**h**) magnetization of integrated MHD pump, (**i**–**l**) photographs corresponding to steps (**a**,**d**,**f**,**h**), respectively.

The magnetic composite with LIG electrodes and the PDMS channel is then subjected to oxygen plasma at 40 W power for 40 s. The two pieces are then placed in contact with each other, thus bonding them and forming the integrated MHD pump. This pump is then magnetized by exposing it to a 1.6 T magnetic field. The fabrication of the MHD pump is depicted in Figure 2.

## 3. Results

### 3.1. Characterization of LIG Electrodes Transferred to Magnetic Composite

Since the transfer of LIG to magnetic composite had never been done hitherto, it was imperative to investigate the material further to ascertain its electrical properties. LIG structures of differing length to width ratios (aspect ratios) were fabricated, and the resistance of these conductors was measured. These results were then plotted, and from the slope of the obtained characteristics, the sheet resistance of native LIG on PI was found to be 23.9 Ω.

Subsequently following the process detailed in Section 2, these conducting LIG structures were transferred to 75% by weight NdFeB PDMS magnetic composite. The resistance measurements were repeated, and it was observed that there is an increase in sheet resistance of the LIG structures to 28.8 Ω. This slight increase in sheet resistance can be explained by (i) the mechanical stress of the PDMS curing within the porous LIG structure and (ii) slight reduction in thickness of the LIG film as some residue is leftover on the PI substrate even after LIG transfer [27].

**Figure 3:**
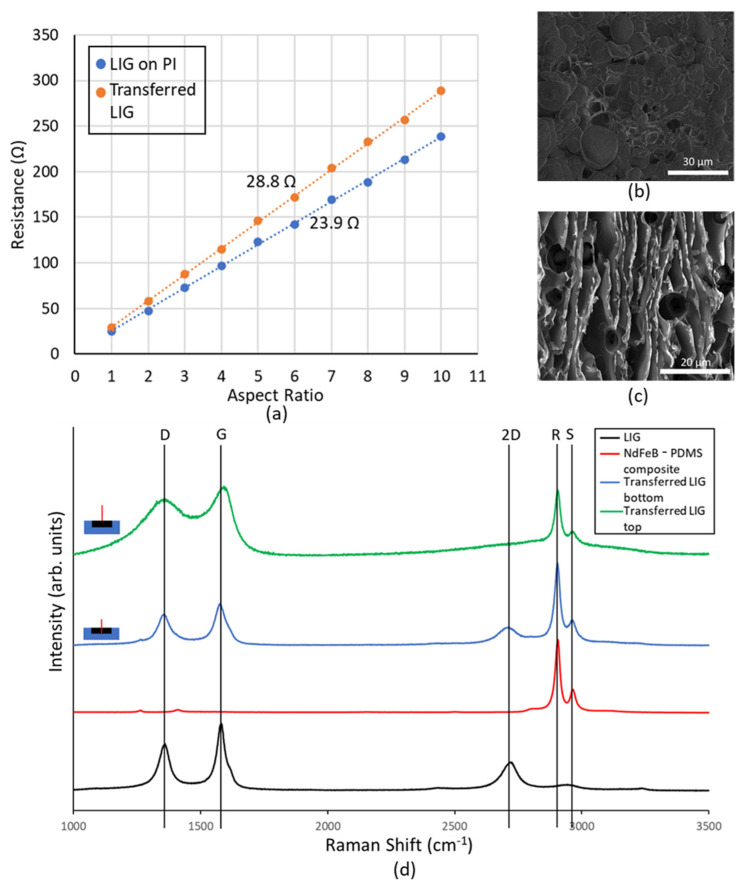
(**a**) Impact of LIG transfer process on sheet resistance, (**b**) SEM image of transferred LIG surface, (**c**) SEM image of native LIG surface, and (**d**) Raman spectrum of transferred LIG, native LIG, and magnetic composite.

The SEM image of the transferred LIG (Figure 3b) shows a far lesser degree of porosity than the native LIG film (Figure 3c). The magnetic composite fills the voids in the LIG, thereby creating a smoother, less porous conductor than native LIG. 

In order to further explore this, the Raman spectrum was obtained for the native LIG, magnetic composite, and transferred LIG at the surface and at the bottom near the magnetic composite LIG interface (Figure 3d). These studies were conducted using a 473 nm Nd:YAG laser, and the spectrum was obtained between wavelength shift values of 1000 cm^−1^ and 3500 cm^−1^. Native LIG shows a spectrum with pronounced D, 2D, and G peaks, which are attributed to the following reasons: (i) at a wavelength shift of ~1350 cm^−1^, the D peak is observed due to the presence of defective and bent sp2 bonds; (ii) at a shift of ~1580 cm^−1^, the G peak occurs due to the presence of doubly degenerate E2g mode; and (iii) the 2D mode occurs at a shift of ~2700 cm^−1^ due to second order zone boundary phonons, and it is indicative of graphene formation. This peak is broader than that seen in monolayer graphene due to the presence of multiple randomly stacked graphene layers [17].

The NdFeB-PDMS magnetic composite spectrum does not have these peaks (D, 2D. or G). Instead, there are two prominent peaks at 2890 cm^−1^ and 2943 cm^−1^, which correspond to the symmetrical and asymmetrical stretching of the methyl (—CH_3_) group, prevalent in PDMS [28]. These peaks are henceforth referred to as R and S peaks, respectively. 

The Raman spectrum taken from the top of the transferred LIG shows lack of sharpness in the D and G peaks. Further, the 2D peak is completely missing in the spectrum. Such spectrum has been reported from the bottom of the LIG film, i.e., near the LIG–PI interface [23]. During the process of graphene formation, as the laser beam penetrates deeper into the PI film, its energy falls and eventually becomes insufficient to cause the photothermal reaction that creates graphene. Thus, at the interface between LIG and PI, conductive amorphous carbon is formed, and it is this material whose Raman spectrum is observed at the bottom of the LIG film. The LIG transfer process flips the vertical order of the native LIG film. Thus, the material at the top of the native LIG film is at the bottom of the transferred LIG film and vice versa. This is further proven by the cross-sectional Raman spectrum of transferred LIG film taken at the bottom (i.e., near LIG magnetic composite interface), which shows distinct D, 2D, and G peaks. 

The transferred LIG spectra obtained from top and bottom of a transferred LIG sample both exhibit prominent R and S peaks, which is indicative of the permeation of magnetic composite material through LIG pores. This explains how the PDMS channel bonds to the LIG electrode material when treated with oxygen plasma. Unlike PDMS, native LIG does not have silanol groups, which are needed to facilitate the oxygen–plasma bonding process [29]. However, since the transferred LIG is filled with PDMS, it is possible to bond the PDMS channel and the magnetic composite with transferred LIG to create an integrated MHD pump.

### 3.2. Integrated MHD Pump Characterization

Silver epoxy (electron microscopy sciences, EMS #12642-14) is used to connect the LIG electrodes of the MHD pump to insulated copper wires for connecting to the electrical supply needed to drive the pump. Saline solution, similar in salinity to sea water, prepared by mixing NaCl in deionized water in 3.5% concentration, was chosen as the working fluid for the integrated MHD pump. Green fluorescent microbeads (Cospheric UVPMS-BG-1.025, diameter 32–38 μm) were first coated with surfactant (Tween 20) and then incorporated in the saline solution in 1:1000 ratio by weight to facilitate flow tracking. 

A low-noise current source (Keithley 6220) was used to drive the MHD pump. This current source itself provides the measured voltage, and a microscope (Leica M125C) was used to monitor the flow of the particles in the pump. 

The current applied was varied from 50 to 300 μA in steps of 50 μA; the corresponding voltage was noted from the source. In order to measure the flow rate, video was recorded using the microscope, and the time needed by the particles to move through a distance of 100 μm was measured. Higher current results in evolution of bubbles from the electrodes disrupting the flow produced by the MHD pump.

**Figure 4:**
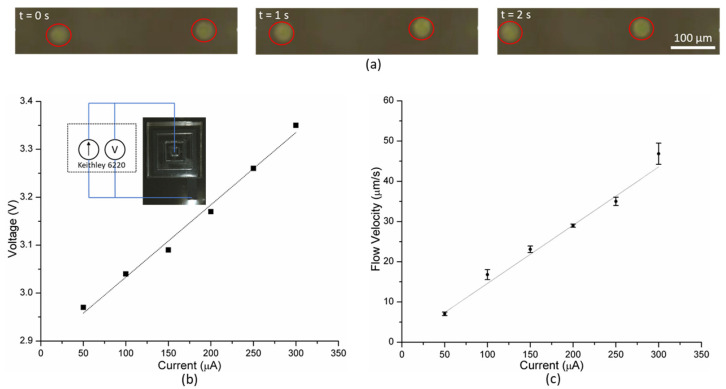
(**a**) Time lapse showing fluid motion in the integrated MHD pump, (**b**) V vs. I; the inset shows the setup used to drive the MHD pump, and (**c**) flow vs. current characteristics of MHD pump.

When current is applied, Lorentz force acts on the saline solution propelling the fluid. This causes the suspended fluorescent microbeads to move (for video, see [30]), which is recorded by the microscope as shown in Figure 4a. The flow velocity of the fluid increases with the applied current. The flow velocity vs. current plot (Figure 4c) can be fitted linearly with a high degree of confidence (R^2^ = 0.98), which agrees well with the theoretical expression (Equation (2)). The current voltage relationship in the operational regime of the MHD pump is linear (Figure 4b). Thereby, the MHD pump can provide higher flow velocity at the expense of greater power consumption.

At a current higher than 300 μA, bubbles were observed in the channel which resulted in inconsistent, disruptive flow. While such behavior could potentially be exploited to facilitate mixing of fluids, it is undesirable for pumping fluids. Thus, the integrated MHD pump produces peak average flow velocity of 46.8 μm/s without any visible bubbles in the channel, when driven by 300 μA.

## 4. Discussion and Conclusions

In this paper, the transfer of LIG to a flexible magnetic composite was demonstrated for the first time. The process outlined herein provides a facile method to fabricate conducting structures on flexible magnetic substrates, which can be useful for other applications as well. The transfer of LIG from PI to the magnetic composite results in a slight degradation in conductivity of the LIG film, with an increase in sheet resistance of ~20%. The transferred LIG film is much smoother and less textured than the native LIG film. This surface smoothness is a key requirement to ensure proper bonding with the PDMS channel for forming the integrated MHD pump. For some applications, particularly energy storage and catalysis, the high surface area of LIG is quite beneficial, and in such cases, it is better to use native LIG on PI rather than released LIG.

The Raman spectrum of the transferred LIG surface indicated the presence of both the LIG film as well as the magnetic composite, which permeated through it during the fabrication process. The presence of NdFeB-PDMS composite on the surface allows the formation of silanol groups on the surface and bonding to the PDMS channel via oxygen plasma treatment.

This is the first ever fully integrated MHD pump with the magnetic-flux source itself serving as the substrate. Just like a conventional MHD pump, the flow rate varies linearly with current. There is an upper bound on the flow rate that can be produced by this pump imposed by the threshold current for bubble formation. This pump compares favorably against other MHD pumps utilizing bubble mitigation techniques as shown in Table 1.

The conductive film transfer method outlined in this work can be used to create conductive traces on PDMS composites without impacting their ability to bond with PDMS or glass. Further, the LIG film electrodes can be functionalized using different chemical groups [23,31], which can allow for its usage as sensing electrodes in lab-on-chip systems. Thereby, it is possible to create various components necessary for lab-on-chip operation such as pumps, mixers, and sensing electrodes using transferred LIG film.

## Figures and Tables

**Table 1:** Flow rate and power consumption of different MHD pumps with bubble mitigation.

Ref.	Bubble Mitigation Technique	Flow Rate (µL/min)	Power (mW)
[15]	Synchronous AC magnetic field and drive current	18.3	8924
[18]	Redox Solution	12.5	1
[19]	Frit structures for bubble isolation	0.5	75
This Work	Low drive current	28.1	1.005

## Data Availability

The data presented in this study is available on request from the authors.

## Supplementary Materials

The following are available online at https://www.mdpi.com/article/10.3390/polym13071113/s1, Figure S1: Magnetization curve of the 75% NdFeB-PDMS magnetic composite material. The inset shows microscopic image of the magnetic composite material. Figure S2: Stress-Strain relationship of PDMS and 75% NdFeB-PDMS magnetic composite.
