# Supplementary material for: Integrated Magnetohydrodynamic Pump with Magnetic Composite Substrate and Laser-Induced Graphene Electrodes
Source: Polymers (Basel). 2021 Apr 1;13(7):1113. doi: 10.3390/polym13071113 (PMC8037410; doi:10.3390/polym13071113)
Supplement: Supplementary file 1 [file polymers-13-01113-s001.pdf]

Supplementary Material

Molycorp MQP-16-7FP NdFeB magnetic micropowder is polydisperse with an irregular morphology and a mean particle size of 5 μm. When incorporated in Sylgard® 184, NdFeB-PDMS magnetic composite material is formed. The microscopic image of the magnetic composite (fig. 1 inset) clearly shows the irregularly shaped NdFeB microparticles, embedded in the PDMS matrix.

The magnetization curve of the magnetic composite is obtained using a vibrating sample magnetometer. At 300 K, the magnetic field was swept from 16 kOe to −16 kOe, while oscillating the sample at 83 Hz with an amplitude of 1 mm. The resultant magnetization curve is shown in Figurer S1. Hard magnetic behavior is observed with a high coercive field (~ −5.8 kOe). This is very desirable as the magnetic composite will resist demagnetization in the presence of weak stray magnetic fields. The magnetic composite exhibits a remanent magnetization of 0.28 T.

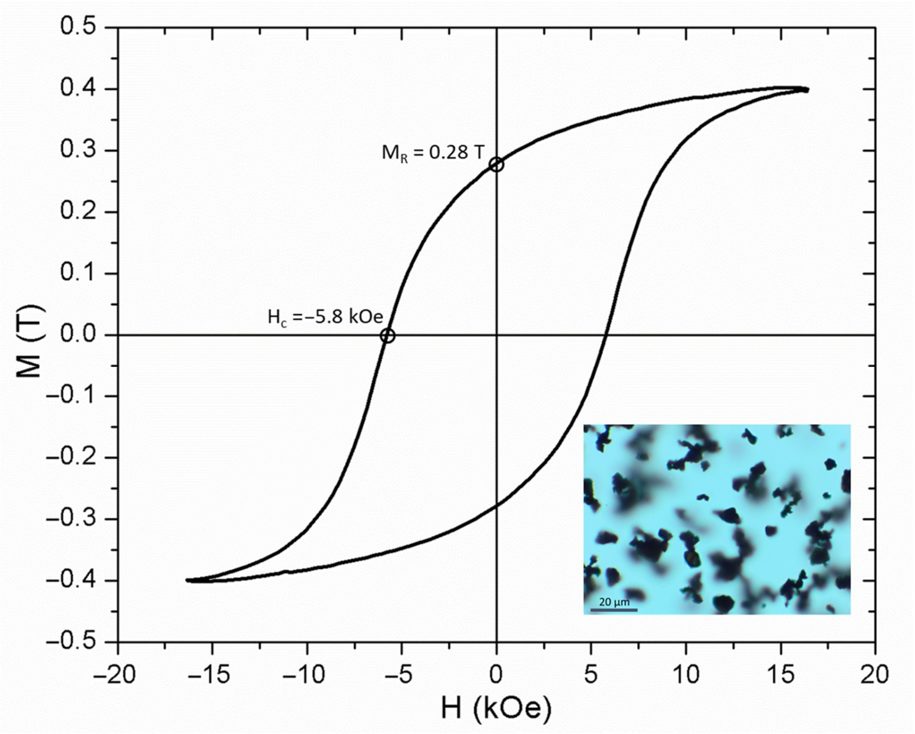

Figure S1 Magnetization curve of the 75% NdFeB-PDMS magnetic composite material. The inset shows microscopic image of the magnetic composite material.

The magnetic composite and pure PDMS were also tested using an extensiometer (Instron 5966). The materials were subjected to an increasing tensile strain and both the stress and strain were recorded. The stress strain relationship for both the pure PDMS as well as the magnetic composite can be fitted linearly (Figure S2). The magnetic composite exhibits a Young's modulus of 12 MPa, which is 8 times greater than that shown by pure PDMS

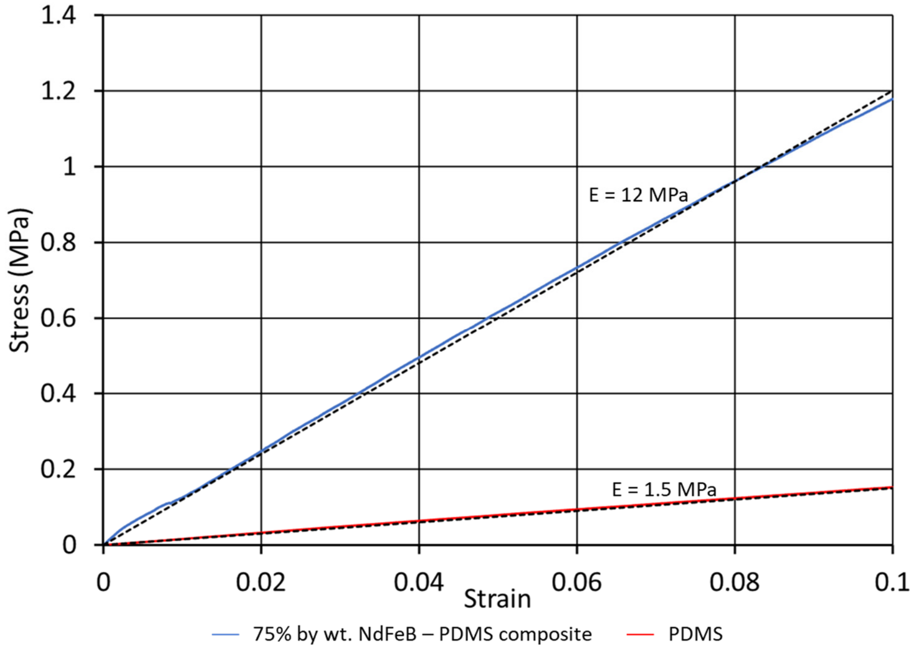

Figure S2 Stress-Strain relationship of PDMS and 75% NdFeB-PDMS magnetic composite
